# ASMNet: Action and Style-Conditioned Motion Generative Network for 3D Human Motion Generation

**DOI:** 10.34133/cbsystems.0090

**Published:** 2024-02-06

**Authors:** Zongying Li, Yong Wang, Xin Du, Can Wang, Reinhard Koch, Mengyuan Liu

**Affiliations:** ^1^ School of Artificial Intelligence, Chongqing University of Technology, Chongqing, China.; ^2^ Multimedia Information Processing Laboratory at the Department of Computer Science, Kiel University, Kiel, Germany.; ^3^ Advanced Institute of Information Technology (AIIT), Peking University, Hangzhou, China.; ^4^ Hangzhou Linxrobot Co. Ltd., Hangzhou, China.; ^5^ National Key Laboratory of General Artificial Intelligence, Peking University, Shenzhen Graduate School, Shenzhen, China.

## Abstract

Extensive research has explored human motion generation, but the generated sequences are influenced by different motion styles. For instance, the act of walking with joy and sorrow evokes distinct effects on a character’s motion. Due to the difficulties in motion capture with styles, the available data for style research are also limited. To address the problems, we propose ASMNet, an action and style-conditioned motion generative network. This network ensures that the generated human motion sequences not only comply with the provided action label but also exhibit distinctive stylistic features. To extract motion features from human motion sequences, we design a spatial temporal extractor. Moreover, we use the adaptive instance normalization layer to inject style into the target motion. Our results are comparable to state-of-the-art approaches and display a substantial advantage in both quantitative and qualitative evaluations. The code is available at https://github.com/ZongYingLi/ASMNet.git.

## Introduction

Current human motion generation tasks aim for highly realistic and natural motions but fall short of meeting the final requirements for direct application in the video games and film industries. Because of the diversity of human motion, encompassing not only simple actions but also stylistic features that convey a character’s personality, emotions, and age. Therefore, when using existing research on human motion generation, it is important for the generated motion to satisfy criteria for both motion content and character motion style. However, there is a scarcity of available datasets containing diverse motion style. Modifying captured motions while preserving the motion content to match a particular style allows for motion data recycling. The generated motion can greatly enhance the motion dataset and be used in downstream tasks, e.g., human behavior recognition [1–7] and robot biomimicry research [8–10]. This approach enables the reuse of motion data while meeting character motion style requirements and reducing the cost of commercial applications.

**Fig. 1.**
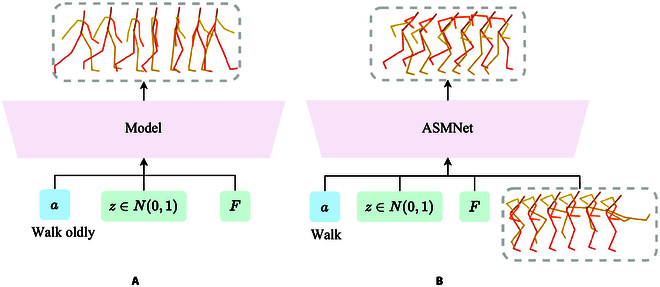
Previous work (A) mostly focused on generating motions based on action label *a*, duration *F*, and latent vector *z*, with the desired style incorporated within the action labels. In our approach (B), we include real-world motions to incorporate specific motion styles in the generation of motions. This enables the model to learn abstract style features that cannot be easily described in text, which are reflected in the generated motions.

Existing works such as MotionCLIP [11] and Motion Diffuse [12] have not only focused on motion generation but also recognized the impact of motion styles on generated results. However, they merely acknowledge the impact of motion styles without emphasizing style features, and simply expect the model to generate stylized motion clips based on the textual descriptions of motion styles (Fig. 1A). Motion style is abstract and cannot be accurately described semantically. Consequently, these approaches only allow for rough generation and fail to exhibit distinct style traits. In addition, the generated motion appears lifeless based on the visual results. It differs from the actual motion effects of people with the same style in real life and lacks distinctive characteristics.

To address the above problems, we attempt to develop a unified model capable of generating human motion sequences that not only conform to the given action categories but also integrate real human motion sequences as style inputs (Fig. 1B). We extract style features from real human motion sequences. Due to the excellent performance of spatial temporal Transformer in tasks such as human motion prediction [13], human motion estimation [14], and human motion recognition [15,16], we also apply the spatial temporal Transformer to the task of human motion generation based on action labels. Our approach not only focuses on extracting motion features for each frame in the sequence but also emphasizes feature extraction between different joints in the same frame. We propose a spatial temporal extractor based on Transformer to extract motion features. By using motion clips corresponding to the action category and providing real motion clips as style sources during training, we enable the model to generate motion sequences that are both natural in motion and exhibit distinct style characteristics during the inference phase. For instance, animation designers in the video game and film industry can be facilitated by using an existing segment of character animation with a distinct style to generate character animation performing different actions within that style.

Our contributions are threefold: (a) We aim to address the problem of three-dimensional (3D) motion generation based on action labels while maintaining explicit motion styles throughout the process. To the best of our knowledge, our approach is the first to address such a problem. (b) We introduce ASMNet, a novel network capable of generating human motion with distinct styles. (c) In our experiments, we perform a thorough ablation study on the model components and outperform state-of-the-art results on the HumanAct12 dataset [17] and the Xia dataset [18].

## Related Work

### Human motion generation

Motion generation can be divided into two main categories: unconstrained motion generation and conditioned motion generation. Unconstrained motion generation models the entire space of possible motions [19–21]. These methods sample from a distribution, allowing for the generation of diverse motions. However, they lack the ability to control the generation process. Conditioned motion generation tends to generate motion based on given control conditions, which can take various forms such as music [22,23], audio [24], speech [25,26], motion clips [27,28], and text information [17,29]. Research on motion generation conditioned on both motion sequences and text descriptions is particularly important. Cui et al. [27,28] use residual graph convolutional network to capture spatial temporal correlation and rely on history information to generate deterministic single future motion. Yuan and Kitani [30] use learnable mapping functions to map samples from a single random variable (generated by a given historical information sequence) to a set of correlated latent codes for further decoding into a set of correlated future motion sequences. Zhang et al. [31] use novel variational autoencoder (VAE) in human motion prediction. Aliakbarian et al. [32,33] use conditional variational autoencoder (CVAE) to enforce diversity and contextual consistency with historical information for final prediction. Generative adversarial network (GAN) [34–36] and normalizing flows [37,38] were used to develop generative models of human motion. Guo et al. [17] and Petrovich et al. [29] propose to use the CVAE-based framework, which not only performs coupled encoding on labels and motion sequences but also treats action labels as conditions to modulate the latent space, resulting in natural and diverse generated motions. We also use the CVAE framework to build the model ASMNet, where we use action labels as conditions. We propose the spatial temporal extractor based on Transformer to construct the motion encoder and motion decoder. In contrast to [17], we do not need multiview cameras to process monocular trajectory estimates. Additionally, unlike [29], we extract motion features not only from the temporal dimension but also from the spatial dimension. This enhances both the temporal correlations between frames in the motion sequence and the spatial connections between joints within each frame.

### Motion style transfer

Previous work relied on handcrafted features [39–41]. Deep learning-based methods extract style features directly from data. Ma et al. [42] model style variations of individual body parts with latent parameters, controlled by user-defined parameters through a Bayesian network. Xia et al. [18] propose a method for constructing mixtures of autoregressive models online to represent style variations in the current pose and apply linear transformations to control style. However, these approaches have limitations such as instability and long computation time. Many current methods now employ neural networks to extract style features. Holden et al. [43,44] propose a style transfer framework composed of a pretrained motion manifold to supervise content and Gram matrices to represent the style of the motion. Mason et al. [45] introduces a residual block for modeling style. Du et al. [46] present a CVAE with the Gram matrices to construct the style-conditioned distribution. These approaches require a lot of computational time to extract style features through optimization and have a limitation in capturing complex or subtle motion features, making style transfer between motions with significantly different contents ineffective. Aberman et al. [47] apply GAN-based architecture with the adaptive instance normalization (AdaIN), which can transfer style from videos to 3D animations. Considering the work of [47], we opt to use real motion clips as input to extract style features. Furthermore, we employ the AdaIN layer to inject style into human motion.

### Action-conditioned styled motion generation

Based on CLIP [48], Tevet et al. [11] propose MotionCLIP, a Transformer-based motion encoder to extract motion features. The model employs text loss to align the latent space of human motion features with the CLIP text label space and image loss to align the motion feature latent space with the CLIP image latent space. This achieves the goal of unifying the CLIP latent space and the motion latent space. Consequently, it enables the generation of human motion sequences based on text labels. MotionCLIP [11] compared the style effectiveness of generated motion with [47]. Of the total eight motion styles evaluated, three achieved higher audience preference scores compared to the latter. This evaluation method, due to its reliance on user study, is highly subjective and does not provide robust persuasive evidence. Furthermore, the animation results from MotionCLIP indicate that human motion across different styles tends to converge toward the same outcome, lacking clear differentiation among various motion styles.

MotionDiffuse [12] incorporates the denoising diffusion probabilistic model (DDPM) into motion generation to establish a mapping between text and motion. MotionDiffuse provides examples of character motion in different styles. However, the transitions between styles in the animation results are not distinguishable. Tevet et al. [11] and Zhang et al. [12] provide textual descriptions of the desired actions and styles, such as “walk angry.” But Aberman et al. [47] require two motion sequences, providing the content and style. Accurately describing the human motion style is challenging due to its abstract nature. To improve the vividness and realism of the generated motion, we aim to use real motion as style input, aligning them with real-world scenarios.

## ASMNet

**Fig. 2.**
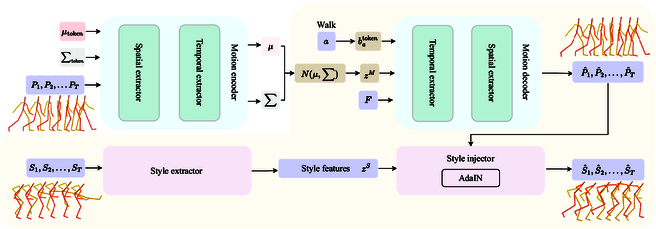
Overview of the proposed ASMNet framework. Training phase: The motion encoder takes a concatenation of motion sequences {*P*_1_, *P*_2_, …, *P_T_*} together with action-specific learnable distribution tokens *μ*_token_ and ∑_token_ as input. It outputs tokens *μ* and ∑ that provide Gaussian distribution parameters, from which a latent vector *z^M^* is sampled. Then, *z^M^*, *F*, and *a* are fed into the motion decoder, which reconstructs the motion information encoded in *z^M^* into a motion sequence $\left\{{\hat{P}}_{1},{\hat{P}}_{2},\ldots ,{\hat{P}}_{T}\right\}$ of length *T* corresponding to action category *a*. To perform style injection, the style extractor extracts the latent style features *z^S^* from the source style {*S*_1_, *S*_2_, …, *S_T_*}. These latent style features are then injected into the generated $\left\{{\hat{P}}_{1},{\hat{P}}_{2},\ldots ,{\hat{P}}_{T}\right\}$ using AdaIN in the style injector. As a result, we obtain the styled sequence $\left\{{\hat{S}}_{1},{\hat{S}}_{2},\ldots ,{\hat{S}}_{T}\right\}$. Test phase (yellow area): Providing only the desired action label *a*, duration *F*, and source style input {*S*_1_, *S*_2_, …, *S_T_*}, we get the target styled motion sequence $\left\{{\hat{S}}_{1},{\hat{S}}_{2},\ldots ,{\hat{S}}_{T}\right\}$.

The whole process is divided into two branches: one for generating motion sequences and the other for injecting style into the motion sequences. Figure 2 illustrates the overall architecture of the proposed method. We employ a CVAE model to accomplish motion generation. The input vector of the motion encoder is a concatenation of human motion sequences *P*_1:*T*_ and action-specific learnable distribution tokens. The spatial extractor in the motion encoder extracts local information between skeletal joints, and the temporal extractor captures global information between frames in the motion sequence. The output of the motion encoder is associated with these tokens, providing Gaussian distribution parameters, and a latent vector *z^M^* is sampled. Next, *z^M^* and a learnable input $b_{a}^{\text{token}}$, determined by the action label *a*, are fed into the motion decoder. This process restores the motion information embedded in the motion latent vector *z^M^* into a human motion sequence ${\hat{P}}_{1:T}$ of length *T*. We employ the learnable distribution tokens for two reasons: first, to fit the sequence-level latent space of human motion based on human motion sequences, enabling the generation of diverse motions given action labels, and second, to assist in capturing spatial temporal motion features more effectively. To achieve style injection, the style extractor extracts latent style features *z^S^* from the source style input *S*_1:*T*_. These latent style features are then injected into the target sequence ${\hat{P}}_{1:T}$ using the style injector, which employs AdaIN layers. The generated sequence ${\hat{S}}_{1:T}$ exhibits a distinct style while adhering to the action category *a*. In the testing phase, only providing wanted action label *a*, durations *F*, and source style input *S*_1:*T*_, we can obtain the target styled action sequences ${\hat{P}}_{1:T}$. During the testing phase, providing the desired action category *a* and the source style input *S*_1:*T*_, we can obtain the target-styled action sequences ${\hat{S}}_{1:T}$. Next, we will provide details on the spatial temporal extractor (“Spatial temporal extractor” section) in motion generation and the process of motion style injection (“Motion style extraction and injection” section). Finally, we will discuss our training process and the associated losses (“Training and loss” section).

### Spatial temporal extractor

**Fig. 3.**
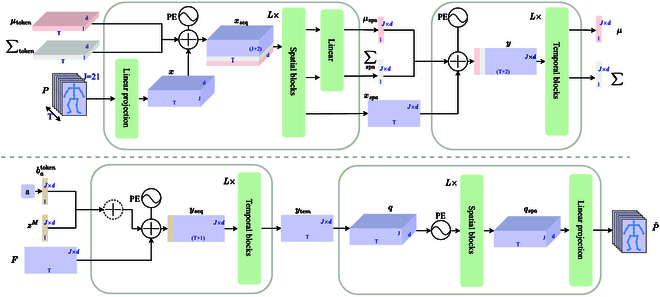
Details about spatial extractor and temporal extractor in motion generation. The figure shows the motion encoder (top) and the motion decoder (bottom), which consist of spatial and temporal extractors (within the gray box) connected in sequential order. The motion encoder takes distribution tokens *μ*_token_ and ∑_token_ and the motion sequence *P*_1 : *T*_ as input. The motion sequence *P*_1 : *T*_ is converted to *x* by a linear layer and then combined with *μ*_token_ and ∑_token_ to form *x*_seq_, which is then subjected to positional encoding before being input to the spatial blocks. At the output of the motion encoder, the part corresponding to the distribution tokens is mapped to the temporal latent space via a linear layer to obtain *μ*_spa_ and ∑_spa_, while the output motion latent tensor is denoted as *x*_spa_. *μ*_spa_, ∑_spa_, and *x*_spa_ are concatenated along the temporal dimension to form the input *y* for the temporal extractor. *y* is passed through the temporal extractor, yielding parameters *μ* and ∑ that represent the latent distribution of the motion features. Using the latent motion representation *z^M^*, a duration *F*, and the action category *a* as input, the motion decoder outputs the generated motion ${\hat{P}}_{1:T}$. *a* is mapped to the motion feature latent space using $b_{a}^{\text{token}}$, then added to *z^M^*, and concatenated with *F*. The resulting tensor *y*_seq_ is subjected to positional encoding before being input to the temporal blocks. The output is the motion features *y*_tem_ in temporal latent space, which is fed into the spatial extractor. Finally, the motion feature *q*_spa_ is reconstructed by a linear projection into the action sequence ${\hat{P}}_{1:T}$.

To generate human motion, we developed a motion encoder *Enc_M_* and motion decoder *Dec_M_* as shown in Fig. 3. We use the spatial extractor to extract the feature embedding from a single frame. First, we map the motion sequence *P*_1:*T*_ ∈ *ℝ*^*T*×*J*×3^ represented by 3D joint position coordinates to a high dimension *d* with a trainable linear projection. Two learnable distribution tokens, *μ*_token_ ∈ *ℝ^d^* and *σ*_token_ ∈ *ℝ^d^*, are provided as input to the spatial extractor during training. During processing in the spatial extractor, we treat each frame as an input and compute the correlations between the 3D position coordinates of the J individual joints within each frame. We consider each token as a joint and concatenate the tokens with the motion embedding, introducing a sense of ordering by sinusoidal positional encoding. Next, the tensor after positional encoding *xseq* is fed into spatial blocks based on the Transformer, and output tokens are then passed through a linear layer to project them up to the temporal embedding dimension (*J* × *d*). The outputs of the spatial extractor are tokens *μ*_spa_, ∑_spa_‍, and the motion vector *x*_spa_. In the temporal extractor, these tokens are concatenated with other encoded frames along the temporal dimension, followed by positional encoding. They then pass through temporal blocks based on Transformer, yielding the distribution parameters *μ* and ∑‍. In summary, our motion encoder may be formally expressed as:

$$\mu ,\sum ‍={\mathrm{Enc}}_{M}\left(\mu_{\text{token}},{\sum ‍}_{\text{token}},P_{1:T}\right)$$ (1)

The motion decoder, denoted as *Dec_M_*, shares a similar architecture with the motion encoder, *Enc_M_*, consisting of a temporal extractor and spatial extractor. To incorporate category information, a learnable bias $b_{a}^{\text{token}}$ is utilized to shift the latent representation toward the motion latent space, which is subsequently added to the latent motion feature *z^M^*. Prior to entering the temporal blocks, positional encoding is applied alongside the action duration, denoted as *F* ∈ *ℝ*^*T*×(*J*×*d*)^, to preserve frame position information. The resulting motion features *y*_tem_ are then passed through the spatial extractor and finally mapped back to a lower dimension using a linear transformation. This process yields the resulting sequence, referred to as ${\hat{P}}_{1:T}$. Our motion decoder can be formally expressed as:

$${\hat{P}}_{1:T}=\mathrm{De}c_{M}\left(a,z^{M},F\right)$$ (2)

where *a* is the action label.

### Motion style extraction and injection

**Fig. 4.**
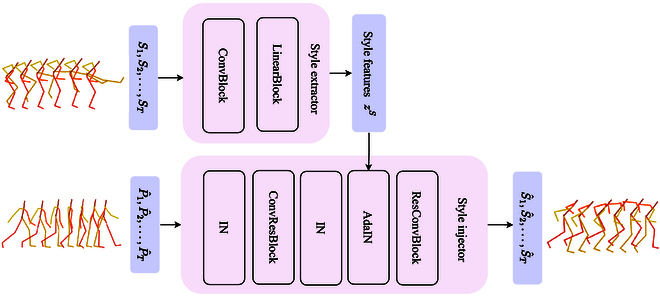
Style injection. Style extractor extracts the style features *z^M^* from the input *S*_1 : *T*_. The style injector first uses instance normalization (IN) to remove the existing style features in ${\hat{P}}_{1:T}$. Finally, *z^S^* are injected into ${\hat{P}}_{1:T}$ using AdaIN. The style injector outputs ${\hat{S}}_{1:T}$.

Our style injection process can be seen in Fig. 4. The source style sequence *S*_1:*T*_ = {*S*_1_, *S*_2_, …, *S_T_*} is entered into the style extractor *Ex_S_*, where *S*_1:*T*_ ∈ *ℝ*^*T*×*J*×3^. The style extractor is stacked by ConvBlock (comprising convolutional layers) and LinearBlock (comprising linear layers), which map *S*_1:*T*_ to a fixed dimensionality independent of sequence length, yielding the latent style encoding *z^S^*. The entire process proceeds as follows:

$$z^{S}=Ex_{S}\left(S_{1:T}\right)$$ (3)

Style injection is achieved through the AdaIN layer in the style injector. The generated action sequence ${\hat{P}}_{1:T}$ is input to the style injector *Ijec_S_*, which consists of ConvBlocks and ResBlocks, which are formed by convolutional layers. There are two types of residual blocks: one to remove the style, and the other to inject the style. Instance normalization (IN) removes the style variations in the motion by normalizing the feature statistics for each channel in each training sample. The motion features after style removal are then injected with action style using AdaIN. AdaIN is the key to successful style deformation. This normalization technique injects the style from ${\hat{S}}_{1:T}$ to the input ${\hat{P}}_{1:T}$ by adjusting the global distribution statistics as:

$$\text{AdaIN}\left({\hat{P}}_{1:T},z^{S}\right)=\sigma \left(z^{S}\right)\left(\frac{{\hat{P}}_{1:T}-\mu \left({\hat{P}}_{1:T}\right)}{\sigma \left({\hat{P}}_{1:T}\right)}\right)+\mu \left(z^{S}\right)$$ (4)

where *μ* and *σ* represent the channel-wise mean and variance, respectively. Finally, the motion features are mapped back to the same dimension as the input using the corresponding ConvBlock in motion injector *Ijec_S_*, resulting in the styled action sequence ${\hat{S}}_{1:T}$. The general process of style injection is as follows:

$${\hat{S}}_{1:T}=\mathrm{Ije}c_{S}\left({\hat{P}}_{1:T}|z^{S}\right)$$ (5)

### Training and loss

Given not only a ground-truth pair consisting of human motion *P*_1:*T*_ and action category *a* but also source style motion *S*_1:*T*_ and same style motion $S_{1:T}^{\text{same}}$, we compare (a) reconstruction loss, (b) KL divergence loss, and (c) content preserving loss.

#### Reconstruction loss

Since ASMNet serves two purposes, our reconstruction loss is utilized in both aspects. First, it is used in action generation. We compare the real motion sequence with the generated sequence based on the action label, using the L2 loss. Second, it is employed in style injection. We compare the real motion sequence with the motion sequence after style injection, using the L2 loss.

#### KL divergence loss

To regularize the latent space, we utilize class-specific distribution tokens in constructing the motion latent space and encourage the distribution to be similar to a normal distribution $\psi =N\left(0,1\right)$. Thus, we obtain the terms:

$$L_{\mathrm{KL}}=\mathrm{KL}\left(N\left(\mu ,\sum ‍\right),\psi \right)$$ (6)

#### Content preserving loss

To ensure that the style features injected into $\hat{P}$ have distinguishable style properties, we not only use the source style input *S* but also select motion sequences *S*^same^ from the dataset *M* that have the same style as *S*. We compute the L1 norm between the results obtained by injecting *S* and *S*^same^ into the motion.

$$L_{\mathrm{con}}=E_{S,S^{\text{same}}∼M}∥\mathrm{Ije}c_{S}\left(S|Ex_{S}\left(S^{\text{same}}\right)\right)-S{∥}_{1}$$ (7)

## Experiments

We implemented ASMNet in PyTorch and conducted the experiments on a PC equipped with an NVIDIA Quadro RTX 5000. The motion encoder and motion decoder in ASMNet utilize spatial and temporal extractors with four blocks, respectively. The latent vector dimension is set to 48 for both. We trained the entire model using the AdamW optimizer with a fixed learning rate of 1 × 10^−4^ and a batch size of 20. The motion generation branch is trained for 200 epochs, while the style injection is trained for 2,000 epochs.

In the subsequent section, we first show the details of the used benchmark dataset and evaluation metrics (“Datasets and evaluation” section). We show the quantitative and qualitative comparison results with the state-of-the-art methods (“Comparison with state-of-the-art results” section). Finally, we analyze the main components of our method (“Ablation studies” section).

### Datasets and evaluation

The dataset used to train ASMNet is Xia dataset [18], a motion capture dataset widely used in style research. The complete database includes six actions (walk, run, jump, kick, punch, and transitions) and eight styles (neutral, proud, angry, depressed, strutting, childlike, old, and sexy). There are 11 min of motion in the database. It was captured with a Vicon optical system at 120 Hz, resulting in roughly 79,000 individual frames. In our processing, motions are retargeted to a single 21-joint skeleton with the same skeletal topology as the CMU (Carnegie Mellon University) motion data. The dataset was split into training and testing sets at a ratio of 0.85:0.15. To evaluate the performance of ASMNet in action generation, we utilized the widely recognized dataset HumanAct12 [17]. HumanAct12 comprises 1,191 motion clips and 90,099 frames in total. All motions are organized into 12 action categories (warm up, walk, run, jump, drink, lift dumbbell, sit, eat, turn steering wheel, phone, boxing, and throw).

We employed the same evaluation metrics as Action2motion [17] to assess the generated motion based on the action category. These metrics include FID (Fréchet inception distance), accuracy, diversity, and multimodality. We followed the same approach as Action2Motion, training a standard recurrent neural network (RNN) action recognition classifier using the training set for Xia dataset [18], and use its final layer as the motion feature extractor. For HumanAct12 [17], we directly use the provided recognition models of Action2Motion [17] that operate on joint coordinates. Subsequently, FID is calculated by comparing the feature distribution of generated motions with that of the real motions. We use the RNN action recognition classifier to classify motion, and calculate the overall recognition accuracy. Diversity measures the variance of the generated motions across all action categories. Two subsets of equal size *S_d_* are randomly selected from a set of all generated motions from different action types. Their respective sets of motion feature vectors {*v*_1_, …, *v_S_d__*} and {*v*′_1_, …, *v*′*_S_d__*} are extracted. The diversity is defined as:

$$\text{Diversity}=\frac{1}{S_{d}}\sum_{i=1}^{S_{d}}{\left\|V_{i}-V_{i}^{\prime }\right\|}_{2}$$ (8)

Multimodality measures how much the generated motions diversify within each action type. Suppose we are given a set of motions with *C* action types. For *c*_th_ action, two subsets of equal size *S_l_* are randomly selected from generated motions, and then two subsets of feature vectors {*v*_*c*,1_, …, *v*_*c*,*S_l_*_} and {*v*′_*c*,1_, …, *v*′*c*_,*S_l_*_}. The multimodality is formalized as:

$$\begin{array}{c} \text{Multimodality}=\frac{1}{C\times S_{l}}\sum_{c=1}^{C}\sum_{i=1}^{S_{l}}{\left\|v_{c,i}^{\prime }-\right\|}_{2} \end{array}$$ (9)

Due to the subjective nature of commonly used human evaluation methods, which can vary among individuals, we employ quantitative evaluation. Similar to [49], the following metrics are computed to evaluate the generated stylized motion sequences: content recognition accuracy (CRA) and style recognition accuracy (SRA). For a fair comparison, we used the same recognition network [50] as for [49] to identify the action category and style category of the generated stylized motion $\hat{S}$.

### Comparison with state-of-the-art results

We first compare motion generation performance based on action labels. Previous work includes Action2Motion [17] and ACTOR [29]. We compared ASMNet with these methods in the Xia dataset [18] and HumanAct12 [17] dataset, as shown in Tables 1 and 2. We also compare with other baselines implemented by Action2Motion by adapting other works [35,36]. Both ACTOR and ASMNet achieve significantly lower FID scores compared to other methods in the HumanAct12 dataset, implying a stronger resemblance between the generated motion distributions and the distributions observed in real human motion. Moreover, ASMNet outperforms ACTOR in terms of accuracy and multimodality, indicating that ASMNet generates more realistic action sequences closely resembling real human motion. In the Xia dataset, our results also outperform previous work in terms of FID, accuracy, and multimodality while maintaining a high level of diversity, showing the superiority of our approach. We employ the spatial temporal extractor to extract motion features and utilize multiple losses for reconstruction, rather than relying on a GRU block and using an autoregressive design like Action2motion. By extracting latent information between skeletal joints, we also incorporate more local skeletal features that help generate better motion sequences than ACTOR.

**Table 1.** Compare with the state of the art in motion generation. We compare with recent work ACTOR and Action2Motion on HumanAct12. Due to differences in implementation (e.g., random sampling, using zero shape parameter), our metrics of real data and ACTOR (Real*, ACTOR*) differ slightly from those in [17,29]. † denotes the baselines implemented by [17,29].

HumanAct12 [17] dataset				
Method	FID↓	Accuracy↑	Diversity→	Multimodality→
Real [17]	0.09^±0.01^	99.7^±0.1^	6.85^±0.05^	2.45^±0.04^
Real*	0.02^±0.00^	99.4^±0.03^	6.85^±0.04^	2.60^±0.01^
CondGRU ([17]†)	40.61^±0.14^	8.00^±0.2^	2.38^±0.02^	2.34^±0.04^
Two-stage GAN [35] ([17]†)	10.48^±0.09^	42.1^±0.6^	5.96^±0.05^	2.81^±0.04^
Act-MoCoGAN [36] ([17]†)	5.61^±0.11^	79.3^±0.4^	6.75^±0.07^	1.06^±0.02^
Action2Motion ([17]†)	2.46^±0.08^	92.3^±0.2^	7.03^±0.04^	2.87^±0.04^
ACTOR ([29]†)	0.12^±0.00^	95.5^±0.8^	6.84^±0.03^	2.53^±0.02^
ACTOR* [29]	0.17^±0.00^	94.9^±0.82^	6.85^±0.04^	2.55^±0.01^
Ours	0.09^±0.00^	96.8^±0.40^	6.84^±0.04^	2.59^±0.02^

**Table 2.** Compare with the state of the art in motion generation. We compare with Action2Motion [17] and ACTOR [29] on Xia dataset [18]. We use the architecture in [17,29] and show the results. The performance improvement with our model shows a significant gap with Action2Motion and ACTOR.

Xia [18] dataset				
Method	FID↓	Accuracy↑	Diversity→	Multimodality→
Real	0.01^±0.00^	96.7^±0.09^	5.70^±0.14^	1.01^±0.05^
Action2Motion [17]	2.49^±0.01^	61.5^±0.03^	5.56^±0.05^	2.32^±0.6^
ACTOR [29]	0.27^±0.00^	89.9^±2.07^	5.56^±0.07^	1.95^±0.12^
Ours	0.17^±0.00^	96.6^±0.71^	5.52^±0.08^	0.97^±0.04^

Moreover, in addition to motion generation, we incorporate a separate branch to inject specific motion styles into the generated motion, which is absent in current motion generation models. Therefore, we compared our approach with methods specifically designed for style research, such as [43,47]. As shown in Table 3, our method is comparable to Motion Puzzle [49] in SRA but surpasses previous methods [43,47,49] by a large margin in CRA, indicating that the motion generated by ASMNet maintains distinct style characteristics while achieving higher accuracy in recognizing the content of the actions, resulting in more realistic human-like motion.

**Table 3.** Comprare with the state of the art in style transfer. We compare with the recent work of [43,47,49] on Xia [18] dataset. Note that due to implementation differences, our metrics of the ground truth real data (Real*) are slightly different than the ones reported in [49]. † denotes the baselines implemented by [49].

Xia [18] dataset		
Method	CRA↑	SRA↑
Real	96.04^±0.00^	90.24
Real*	95.35^±0.00^	90.70
Amberman [47]†	29.09^±1.29^	41.97^±2.01^
Holden [43]†	38.93^±2.09^	41.92^±1.77^
Motion Puzzle [49]†	29.83^±1.35^	54.94^±2.09^
Ours	74.12^±0.00^	52.94^±0.00^

**Fig. 5.**
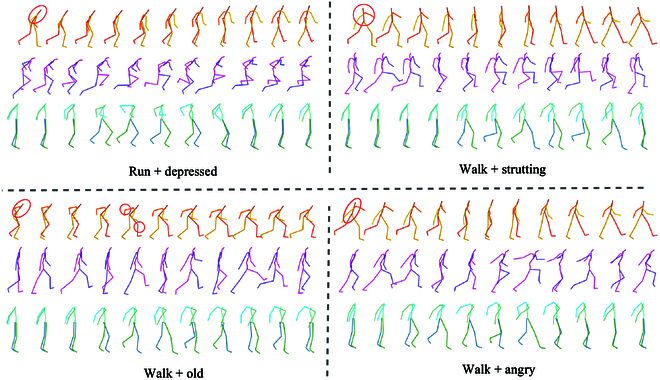
We compare the generated styled motion animation results with ASMNet (orange), MotionCLIP [11] (purple), and MotionDiffuse [12] (green) for the action “walk” under four different motion styles (depressed, strutting, old, and angry).

In addition to quantitative analysis, we also provide visualizations of the generated motion for comparison with motionCLIP [11] and motionDiffuse [12] in Fig. 5. We choose the “walk” action to demonstrate four different styles. It is evident that although all individuals perform the action of walking, there are significant differences in the posture and amplitude of the movements due to the different styles. In Fig. 5, it can be seen that our motion sequences show the most pronounced and visually appealing style variations. In contrast, motionCLIP [11] and motionDiffuse [12] show similar results regardless of style changes. Both quantitative and qualitative analyses confirm the excellent performance of ASMNet in motion generation with motion style.

**Fig. 6.**
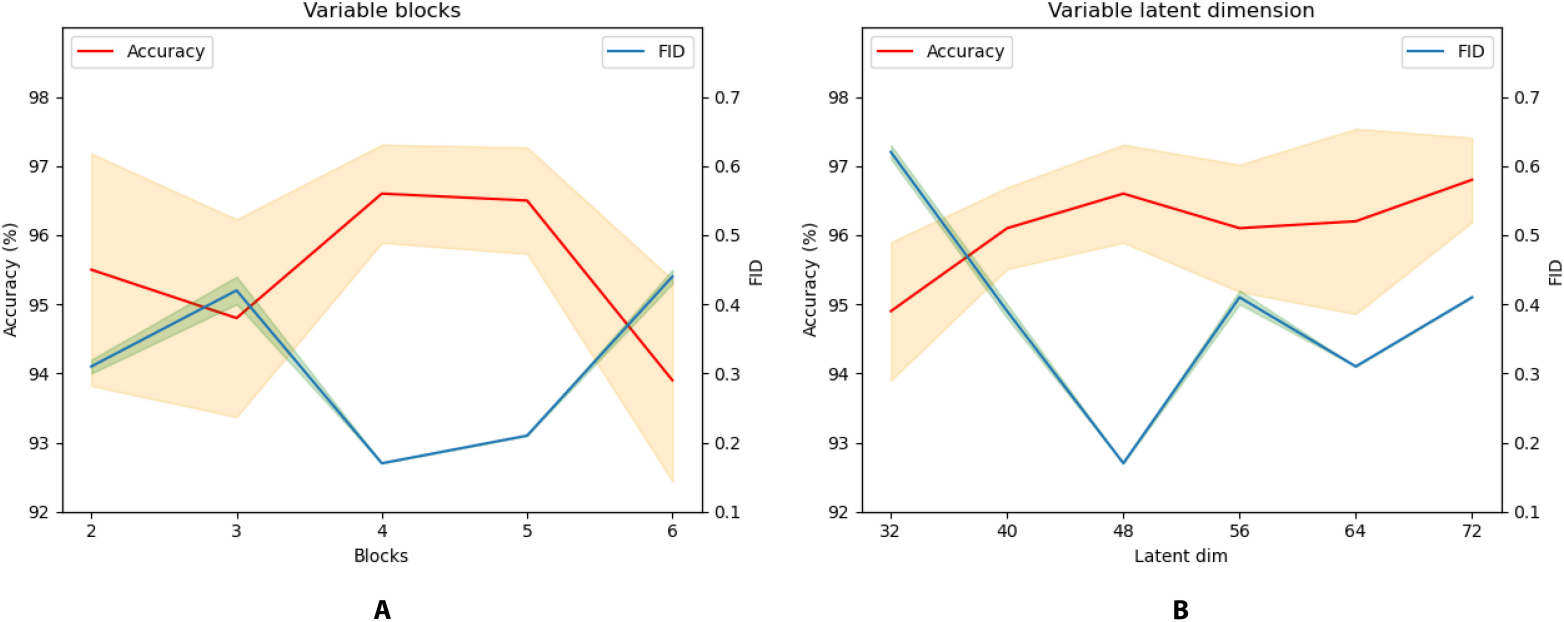
Ablation over the number of blocks in the spatial temporal extractor (A), when fixing the latent dimension in the spatial extractor to 48. Ablation about the latent space dimension *d* (B) mentioned in motion encoder and motion decoder, when fixing the blocks in spatial temporal blocks to 4. The dashed area represents the fluctuation error of the metrics.

### Ablation studies

In this section, we evaluate the impact of several components of our framework in a controlled setting. First, we ablate several architectural alternatives. We replace the spatial temporal extractor in the motion encoder and motion decoder with the fully connected autoencoder, GRU, and Transformer separately in Table 4A to C. We see that our ASMNet outperforms the methods based on fully connected layers and GRU by a large margin. It also outperforms the Transformer-based methods. The spatial temporal extractor in our ASMNet excels at modeling the temporal evolution of human motion, in contrast to fully connected layers and GRU, providing more accurate modeling capabilities for human movement. Moreover, the spatial temporal extractor has the unique capability to capture the local correlation between joints in the human skeleton, which evades Transformer.

**Table 4.** Ablation on ASMNet architecture. We replace the architecture of the motion encoder with fully connected layers (A), GRU (B), and Transformer (C). We omit the usage of $b_{a}^{\text{token}}$ in the motion decoder and instead convert the motion action label to one-hot encoding (D). We remove the input of *μ*_token_ and ∑_token_ in motion encoder (E) and take only the real motion as input.

Xia [18] dataset				
Method	FID↓	Accuracy↑	Diversity→	Multimodality→
Real	0.01^±0.00^	96.7^±0.09^	5.70^±0.14^	1.01^±0.05^
(A) Fully connected	16.81^±0.67^	16.1^±3.60^	4.39^±0.04^	4.30^±0.08^
(B) GRU	4.28^±0.59^	37.2^±4.45^	6.00^±0.12^	5.38^±0.25^
(C) Transformer	0.82^±0.04^	86.0^±2.25^	5.51^±0.14^	2.24^±0.12^
(D) W/o $b_{a}^{\text{token}}$	5.72^±1.39^	46.6^±2.90^	3.69^±0.16^	3.91^±0.18^
(E) W/o *μ*_token_, ∑_token_‍	0.27^±0.00^	93.6^±1.28^	5.39^±0.07^	1.15^±0.09^
Ours	0.17^±0.00^	96.6^±0.71^	5.52^±0.08^	0.97^±0.04^

We also remove the token $b_{a}^{\text{token}}$ that transforms the action category information into the latent motion space. Instead, we convert the action category to the one-hot encoding and input it directly into the motion decoder (Table 4D). We remove the distribution parameters tokens *μ*_token_ and ∑_token_‍, which are input to the motion encoder. We derive the distribution parameters *μ* and *σ* by averaging the output of the motion encoder (Table 4E). As expected, our results in Table 4 outperform other methods, demonstrating the superior structure of ASMNet. One-hot encoding directly inputs the binary encoding of action categories to the motion decoder, failing to capture the deep semantic information of action categories. In contrast, the learnable token $b_{a}^{\text{token}}$ maps action categories to a high-dimensional semantic space, enabling the model to obtain richer semantic information and understand the similarities between different action categories. This facilitates the model’s learning of the complex relationship between action categories and motion sequences, resulting in improved generation of motion sequences that align with specific actions.

Finally, we conduct ablation experiments on the number of spatial blocks and temporal blocks in the spatial extractor and temporal extractor, as shown in Fig. 6A. The results demonstrate that the accuracy of generated motion sequences is highest and the FID score is lowest when the number of blocks is 4, indicating the best performance. We note that the accuracy of the generated motion reaches its maximum when the latent dimension is set to 48, and that the score FID reaches its minimum in Fig. 6B. Therefore, when constructing ASMNet, we set the number of blocks in the spatial temporal extractor to 4 and the latent dimension *d* to 48.

## Conclusion

In this work, we target the motion generation with explicit style conditioned on the action label. We propose a comprehensive model named ASMNet. We extract motion features from both the temporal and spatial dimension by the spatial temporal extractor, which enables us to enhance the accuracy and increase the diversity in motion generation. Moreover, we also note the influence of motion style in the generation process. Instead of using a simple textual representation, we use real motion to provide the style and use AdaIN for style injection, resulting in generated motion sequences with distinct stylistic attributes. We also provided a detailed analysis to assess different components of our proposed approach. The experiments show the superiority of ASMNet, which not only satisfies the motion generation but also incorporates explicit stylistic attributes into the generated motion. Our approach outperforms the state of the art in both quantitative metrics and visual analysis, highlighting its effectiveness. Of course, AMSNet has its limitations, as the current study of motion styles relies on motion capture data, with the challenge of scarce mocap datasets with motion styles. Future work can therefore explore obtaining sufficient motion style data from other forms of data to generate motion with more diverse styles.
